# Ear Thermography as a Candidate Dynamic Index of Sympathetic and Parasympathetic Activity

**DOI:** 10.3390/s26123819

**Published:** 2026-06-16

**Authors:** Wataru Sato, Budu Tang, Koh Shimokawa

**Affiliations:** 1 Psychological Process Team, Guardian Robot Project, RIKEN, 2-2-2 Hikaridai, Seika-cho, Soraku-gun, Kyoto 619-0288, Japan; tang.budu.53s@st.kyoto-u.ac.jp (B.T.); koh.shimokawa@riken.jp (K.S.); 2 Graduate School of Informatics, Kyoto University, Yoshida-Honmachi, Sakyo, Kyoto 606-8507, Japan

**Keywords:** autonomic nervous system, emotional arousal, ear thermography, sympathetic and parasympathetic divisions

## Abstract

Monitoring the activity of the autonomic nervous system, including the sympathetic and parasympathetic divisions, plays a crucial role in studying emotional processing. However, few methods allow the dynamic tracking of parasympathetic activity. Here, we propose a testable hypothesis that ear thermography may serve as a dynamic index of sympathetic and parasympathetic activity, with a time resolution of seconds. Anatomical and physiological evidence suggests that the vascular structures of the ear may be innervated in a region-specific manner by the autonomic nervous system, with the posterior regions (e.g., the helix) predominantly influenced by sympathetic activity and the anterior regions (e.g., the tragus) potentially affected by parasympathetic mechanisms. Recent thermographic studies during emotional film viewing have demonstrated distinct spatial and functional patterns: posterior regions showed a linear negative association between temperature and emotional arousal, consistent with sympathetic vasoconstriction, whereas anterior regions exhibited a non-linear (inverted-U-shaped) relationship, resembling the known non-monotonic characteristics of parasympathetic activity. These findings suggest that ear thermography may be used to assess sympathetic- and parasympathetic-related dynamic processes, although direct evidence remains to be established.

## 1. Introduction

Measurement of autonomic nervous system (ANS) activity plays a crucial role in monitoring emotions. Theoretically, as James [1] and several other researchers [2,3] have proposed, bodily responses, such as ANS activity, may constitute the essence of subjective emotional experience. If this form of subjective–physiological concordance indeed exists, it will provide important clues for those seeking to understand the psychological mechanisms underlying emotions. In practical terms, the physiological signals of ANS activity would be expected to deliver unbiased dynamic measures of subjective emotional states that allow prediction of subsequent behaviors, such as purchasing [4].

The ANS is a component of the peripheral nervous system and is classically divided into two primary divisions, including the sympathetic nervous system (SNS) and the parasympathetic nervous system (PSNS) [5] (however, a possible third division, the enteric nervous system, has been discussed [6,7]). The SNS and PSNS affect nearly every bodily tissue to control cardiovascular and visceromotor functions [5]. They are primarily defined as efferent systems, although their associated nerves also contain afferent fibers that convey visceral sensory input [5]. The efferent pathways are typically organized as two-neuron chains outside the central nervous system; each chain consists of preganglionic neurons, with cell bodies in the brainstem or spinal cord, and postganglionic neurons, with cell bodies in the peripheral ganglia that directly innervate target tissues [7]. Efferent autonomic outputs from the brain are generated and regulated by brainstem circuits within a distributed central autonomic network, in coordination with higher cortical and subcortical regions, including the insula, amygdala, and hypothalamus [8]. Specifically, in the SNS, key premotor regions include the rostral ventrolateral medulla, which provides a major excitatory drive to cardiovascular sympathetic outflow, and the rostral ventromedial medulla, which is more involved in thermoregulatory and cutaneous sympathetic control [9]. In the PSNS, preganglionic neurons originate from the Edinger–Westphal nucleus, the superior and inferior salivatory nuclei, the dorsal motor nucleus of the vagus, and the nucleus ambiguus [10].

To record ANS activity, several measures have been developed (see [11] for a review). A representative measure of SNS activity is electrodermal activity (e.g., the skin conductance response [12]). Such activity mirrors the electrical activation of eccrine sweat glands, which are innervated by the SNS [12], and delivers dynamic information on SNS activity with a time resolution of seconds. A commonly used indirect index of PSNS activity is heart rate variability, which is controlled by both SNS and PSNS activity [13]. After evaluation of heart rate data collected over several minutes (a minimum of 5 min is recommended [14]), the power of the high-frequency spectrum, ranging from 0.15 to 0.4 Hz (or other values when assessing variability within this range), can yield an index of PSNS activity [13]. Although dynamic measures of heart rate variability have been proposed (e.g., using 32 s sliding windows [15]), debate remains regarding the validity of such short-term analyses [16,17]. In brief, researchers can assess SNS and PSNS activity using several different measures.

However, there appear to be few methods that allow high-temporal-resolution tracking of PSNS activity. Such dynamic information on ANS activity could be important because several psychological studies on dynamic ratings have shown that emotional experiences change from moment to moment during the processing of emotional stimuli and diverge from the overall ratings after stimulus presentation [18,19]. It was also shown that such short-term emotional dynamics exhibited unique associations with long-term well- and ill-being, such as the link between large moment-to-moment emotional fluctuations and maladaptive psychological functioning [20]. Several studies have acquired dynamic information on SNS activity using an electrodermal technique (e.g., [21]). In contrast, few works examined dynamic changes (i.e., at time resolutions of seconds) in PSNS activity because appropriate measures are lacking. Such information could be relevant, as the PSNS may play a more nuanced role than previously thought. Although classical research has suggested that the PSNS and SNS operate in an antagonistic manner [22], subsequent studies have shown that they can function in a complementary manner (e.g., in salivary glands, where both divisions promote secretion, with PSNS activation driving fluid secretion and SNS activation enhancing protein release [23]) or a cooperative manner (e.g., in sexual function, where PSNS activity mediates erection and SNS activity mediates ejaculation as part of a coordinated physiological sequence [24]). In the context of emotion, PSNS and SNS activity were traditionally assumed to reflect positive and negative emotions, respectively [22], but subsequent studies demonstrated that the PSNS could be activated during some negative emotions, such as fear and sadness (e.g., [25]; for a review, see [26]). Together, the data suggest that a dynamic measure of PSNS activity would be useful when seeking to understand PSNS function in terms of emotion.

Based on the findings of recent studies [27,28], together with related anatomical and physiological evidence, we hypothesize that infrared ear thermography may provide valuable dynamic indices of SNS and PSNS activity. Previous studies have reported that facial thermal imaging can indicate ANS activity by quantifying thermal signal changes resulting from cutaneous vascular changes and/or subjective emotional states (for a review, see [29]). Specifically, several studies consistently observed that the temperature of the nose tip decreased during the processing of emotional stimuli, possibly due to SNS-mediated vasoconstrictive mechanisms (e.g., [30,31,32,33]). Such temperature changes reportedly occur rapidly, within 2–3 s after stimulus onset [31], and exhibit second-by-second fluctuations [32]. While temperature changes in other facial regions, including the forehead (e.g., [34]) and the periorbital region (e.g., [35]), have also been reported, the results appear inconsistent, possibly due to the lack of clear SNS or PSNS innervation. Such changes in the ear have remained largely unexplored until recently [27,28]. In the following sections, we first describe the background anatomical and physiological data of ANS ear innervation. Next, we summarize the findings. Finally, we conclude with a discussion of future work and the implications thereof.

## 2. Anatomical and Physiological Information Underlying Ear Temperature Change

**Figure 1:**
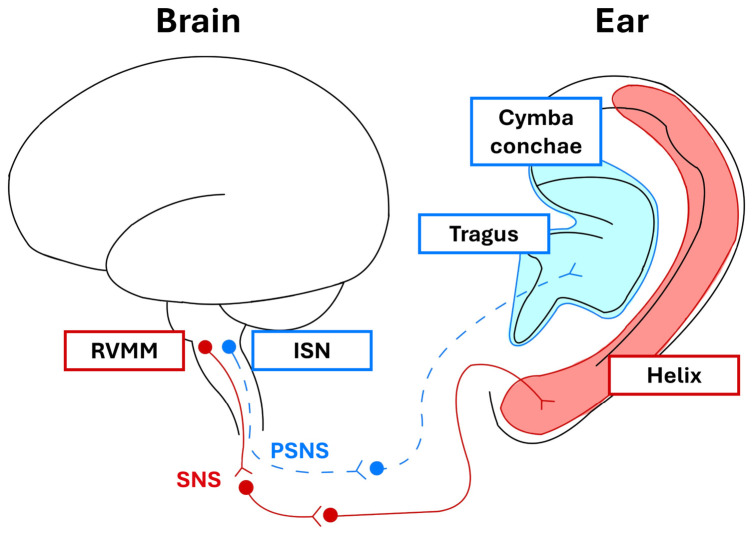
Hypothesized autonomic pathways relevant to regional changes in ear temperature. In the brain, the rostral ventromedial medulla (RVMM) and inferior salivatory nucleus (ISN) are identified as putative central sources that may contribute to sympathetic nervous system (SNS)- and parasympathetic nervous system (PSNS)-related influences of the ear, respectively. In the ear, posterior regions (e.g., the helix) are proposed to be influenced predominantly by the SNS, whereas anterior regions (e.g., the tragus and cymba conchae) may be affected by PSNS-related pathways. The rationale for this view of auricular nervous innervation is derived from [39,42].

Anatomical studies in humans and animals have suggested that the external ear, or auricle, is a unique region with region-specific innervation by both the SNS and PSNS. Our hypothetical autonomic pathways underlying ear temperature changes are presented in Figure 1.

First, cutaneous blood vessels are predominantly under sympathetic neural control, which generally induces vasoconstriction [36], although some evidence suggests that this principle may not apply uniformly across all craniofacial regions, as discussed below. SNS fibers generally innervate blood vessels via perivascular plexuses, forming diffuse neuroeffector junctions [37]. Given the anatomical evidence of a relatively dense arterial distribution across the human helix [38,39], the effects of SNS signals from the rostral ventromedial medulla on cutaneous vascular tone [9] would be particularly pronounced in relatively posterior auricular regions, including the helix. Here, “posterior” refers to a region on the horizontal axis used in anthropometric studies on the ear [40].

Next, human anatomical studies have shown that the relatively anterior ear regions, including the tragus and cymba conchae, are innervated by the auricular branch of the vagus nerve (ABVN), or Arnold’s nerve (e.g., [41]; for a review, see [42]). Ample anatomical and physiological evidence in humans and other animals has indicated that the ABVN is primarily a sensory (afferent) branch of the vagus nerve, which is a major component of the PSNS (for a review, see [42]). However, a recent developmental anatomical study in humans suggested that the ABVN-rich region receives projections from the glossopharyngeal nerve [43]. In this work, researchers examined serial histological sections of 55 human embryos and fetuses to investigate the origin and course of the ABVN. They found that the ABVN initially develops from the glossopharyngeal nerve, which then almost simultaneously builds a connection with the vagus nerve. These findings suggest that the ABVN-rich region contains connections not only to the vagal nerve but also to the glossopharyngeal nerve, at least during the prenatal stage. This notion is consistent with several anatomical findings in human adults (e.g., [44,45]; for a review, see [42]). The glossopharyngeal nerve is a mixed nerve [46] that includes PSNS efferent fibers from the inferior salivatory nucleus [47,48], which constitutes the neural circuit with the nucleus tractus solitarius [49] that plays an integrative role in PSNS activity [50,51]. Several physiological studies in animals have demonstrated that the glossopharyngeal nerve transmits PSNS activation from the inferior salivatory nucleus to induce vasodilation of the facial skin (e.g., [52]; for a review, see [53]). Although most skin blood vessels are regulated by the SNS [36], it has been proposed that several craniofacial structures, including the auricle, receive both SNS and PSNS inputs [51]. An association between the ABVN region of the ear and PSNS activity has also been suggested by studies that tested the effects of electrical stimulation of the ABVN region, known as transcutaneous auricular vagus nerve stimulation. Several studies have shown that stimulation of the tragus induces a shift in heart rate variability toward PSNS predominance (e.g., [54]; for a review, see [55]), although the neural pathway involved remains unclear [56]. Taken together, these data suggest the possibility that anterior ABVN-rich regions may not only send afferent projections through the vagus nerve but also receive PSNS-related influences via the glossopharyngeal nerve and the inferior salivatory nucleus.

Some ABVN-rich regions may also be influenced by SNS and PSNS activity via the trigeminal nerve through its branch of the auriculotemporal nerve [57]. Anatomical studies have suggested that the trigeminal nerve is mainly somatosensory but carries accompanying SNS and PSNS fibers from other sources [58,59]. However, such studies also suggested that the auriculotemporal nerve primarily innervates the anterosuperior region relative to the tragus (for a review, see [60]). Hence, we speculate that the effect of the trigeminal nerve may not be substantial in the ABVN-rich region and may be more evident in the relatively anterosuperior region.

In summary, several sources of evidence suggest that vascular and temperature changes in the subregions of the ear may reflect distinct ANS activation, specifically SNS activation in posterior regions, such as the helix, and PSNS activation in anterior regions, such as the tragus.

## 3. Recent Evidence of Emotion-Related Ear Thermal Changes

Two recent studies have recorded ear thermography data while subjects viewed emotionally evocative films and suggested that ear temperature may indicate the dynamics of SNS- and PSNS-related processes.

In Tang and Sato [27], the researchers acquired unilateral (right) ear thermal images using an infrared thermal camera while 15 participants viewed five emotionally evocative clips that elicited responses ranging from very positive to very negative [32]. After the initial viewing during physiological data acquisition, the clips were shown twice more; participants recalled and dynamically reported valence or arousal ratings using a slider-type affect rating dial [61]. To examine the relationship between ear thermal images and dynamic emotional ratings, a pixel-based analysis for 1 Hz sampled thermal images, controlling the family-wise error rate across the entire image using random field theory, was conducted with statistical parametric mapping [62].

**Figure 2:**
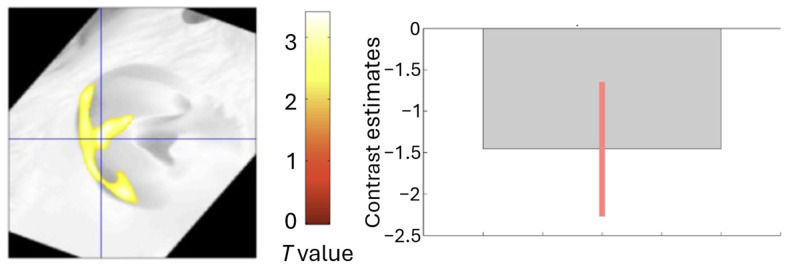
The statistical parametric map derived from Tang and Sato [27], showing the significant negative association between subjective arousal ratings and temperature in the posterior auricular regions, and the contrast estimate plot (with a 90% confidence interval) at the activation focus. The confidence interval provides information regarding the magnitude and precision of the estimated effect and indicates that the association was likely negative at the population level.

The results, corrected for multiple comparisons, revealed a negative linear association between ear temperature and emotional arousal across broad posterior ear regions, including the helix (Figure 2). As heightened emotional arousal is typically associated with increased SNS activation [63,64], which in turn decreases temperature via skin vasoconstriction [36], the result suggests that the temperature decrease is consistent with sympathetic vasoconstrictor activity during emotional arousal. The spatial pattern of the broad posterior ear regions, including the helix, is consistent with the above-suggested notion that SNS influences would be particularly evident in posterior auricular regions, including the helix, in line with human anatomical data [39].

In a follow-up study [28], the same researchers re-analyzed their dataset using machine learning models to investigate nonlinear associations between ear thermal images and self-reported arousal ratings while participants watched emotion-eliciting films. Three models were constructed: a multiple linear regression model using the posterolateral ear regions as regions of interest as a baseline; a random forest model (a representative conventional machine learning approach [65]); and a ResNet-50 convolutional neural network (a representative deep-learning-based machine learning model [66]).

Evaluation of the predictive performance of each model via leave-one-participant-out cross-validation showed that both machine learning models, particularly ResNet-50, outperformed linear regression in terms of predicting emotional arousal dynamics based on ear thermal imaging data. Such results imply that ear temperature–arousal associations are not exclusively linear but also include nonlinear components.

**Figure 3:**
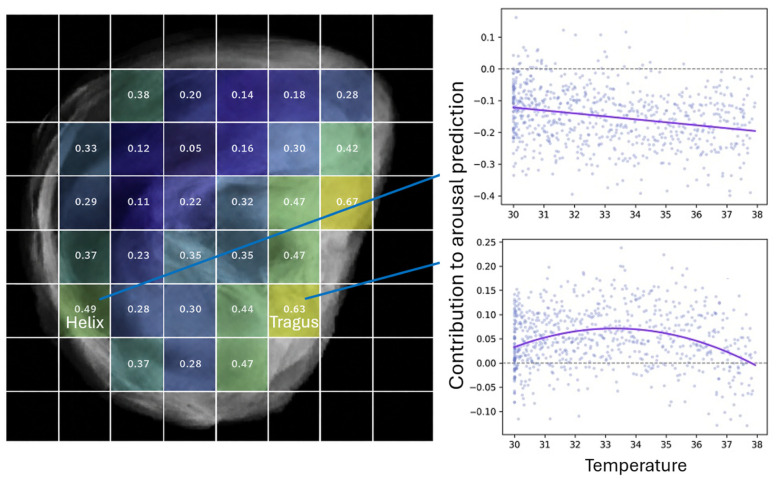
The results of model interpretation by Tang and Sato [28] showing that the helix and the tragus contribute to the prediction of subjective arousal ratings (explaining model behavior, not physiology directly), with negative linear and negative quadratic associations, respectively.

To interpret how ResNet-50 predicted arousal, the spatial attention patterns of the model were explored using gradient-weighted class activation mapping [67]. These methods identify model-attended regions and descriptive patterns of feature contribution, although they cannot provide causal physiological evidence. The model assigned high importance to some specific subregions, including posterior (including the helix) and anterior (including the tragus) regions (Figure 3). Next, to examine how the temperatures of these regions were associated with arousal predictions, Shapley additive explanation values [68] were calculated. The relationships between temperature and arousal predictions were then examined by fitting a series of polynomial regression models, including first- (linear), second-, third-, and fourth-degree functions. To avoid overfitting while allowing for potential nonlinear associations, the relative performance of these models was compared using the Bayesian information criterion, which balances goodness of fit against model complexity. The model with the lowest Bayesian information criterion value was selected as the optimal model. The results showed that, first, the helix exhibited a linear negative relationship between temperature and arousal ratings, consistent with the results of statistical parametric mapping. More importantly, two anterior areas, including the tragus, evidenced negative quadratic (i.e., inverted-U-shaped) associations between temperature and the arousal ratings: local temperature increased as arousal rose to a certain level.

The negative quadratic associations between temperature and arousal ratings in anterior regions, such as the tragus, are notable in the context of the present study. As noted above, the anatomical and physiological findings suggest that the anterior regions are innervated by the PSNS. The negative quadratic relationship that was found may be compatible with the findings of Goldberger et al. [69] who systematically investigated PSNS effects. In this work, researchers examined the relationship between PSNS effects and heart rate variability by pharmacologically manipulating baroreflex activity under a β-adrenergic blockade. They quantified the effects of the PSNS using the time duration between successive heartbeats and evaluated how multiple heart rate variability metrics changed across graded levels of PSNS activation. The results showed that a quadratic model explained the data better than a linear model, revealing a non-monotonic relationship characterized by an initial increase followed by a decrease in variability at higher levels of PSNS effect. Such a nonlinear pattern indicates that physiological responses mediated by the PSNS may exhibit diminishing or even reversed effects at higher levels of activation. The researchers suggested that intense parasympathetic discharges may release sufficient acetylcholine to saturate its effects on the sinoatrial node, thereby reducing the phasic modulation of heart rate and leading to a decrease in heart rate variability. Saturating dose–response patterns for acetylcholine-induced cutaneous vasodilation have also been observed in human skin [70,71]. Therefore, one may speculate that the negative quadratic relationship observed in the anterior region of the ear may reflect a similar nonlinear modulation of peripheral physiological signals by PSNS activity. However, such speculation should be cautious, as Goldberger et al. [69] tested cardiac vagal function rather than auricular temperature regulation. In addition, debate remains regarding the relationship between PSNS activity and heart rate variability [72]. Therefore, we cite it only as a heuristic analogy suggesting that non-monotonic parasympathetic-related physiology may be a plausible explanation for the data, which otherwise do not establish an auricular mechanism.

In summary, these findings suggest that ear thermography may provide dynamic information about SNS- and PSNS-related processes.

## 4. Discussion

Based on recent thermography findings, together with anatomical and physiological data on changes in ear temperature, we propose that ear thermography may serve as a dynamic index of SNS and PSNS activity. We acknowledge that the current evidence is preliminary and there are several limitations. First, the findings were based on a small sample of healthy young Japanese participants, with no independent external replication. Hence, the results should be interpreted as exploratory and hypothesis-generating. Second, experiments were conducted under controlled laboratory conditions with relatively stable ambient temperatures, which may not have fully reflected the thermal noise and environmental variability present in unconstrained real-world settings. Third, only the right ear was measured due to limitations of the apparatus, leaving potential functional hemispheric differences in ANS activity [73]. Fourth, during image preprocessing, a frame-by-frame affine transformation was applied, but it could not remove non-affine distortion caused by motion artifacts. The application of improved preprocessing methods and evaluation of the confounding effects of motion artifacts are warranted. Finally, direct evidence for the anatomical and physiological mechanisms is lacking. Specifically, the interpretation of the nonlinear pattern in the tragus is based on an analogy to the cardiac PSNS pattern [69], and other interpretations (e.g., interactions between the PSNS and SNS, local thermal regulation) may be possible. In addition, the pathway discussion was based primarily on cadaveric anatomical studies, which cannot establish physiological connectivity. However, we considered indirect data to be an adequate basis for a testable hypothesis.

To test the hypothesis empirically, various future studies are warranted. First, it would be useful to simultaneously measure ear temperature and collect data on established physiological indices, such as electrodermal activity and heart rate variability. Correlational analysis could help to determine whether specific auricular regions track SNS and PSNS activity. Second, experimental manipulations that stimulate specific divisions of the ANS could be used to obtain causal evidence on the association between ANS activity and changes in ear temperature. Some studies have proposed that certain tasks (e.g., the cold pressor test, in which one hand is immersed in ice water) primarily activate the SNS, whereas other tasks (e.g., deep breathing) stimulate the PSNS [74]. Finally, SNS and PSNS activity should be experimentally manipulated using pharmacological agents such as atropine (parasympathetic blockade) [75]. Evaluation of how such changes affect auricular temperature patterns could yield causal evidence of SNS and PSNS involvement in ear temperature changes. We expect that the findings of these basic validation studies may demonstrate that ear thermography can discriminate SNS- and PSNS-dominant stages using short-duration data, relative to traditional ANS measures such as heart rate variability. In such studies, it would be important to account for factors identified in previous studies (e.g., functional hemispheric differences and ambient temperature), as well as sex, menstrual cycle phase, and time of day, which may modulate ANS activity [76]. In addition, the field may benefit from the development of an open-science platform for sharing ear thermography datasets, analysis pipelines, and experimental protocols. Such a framework could improve reproducibility, promote independent validation of proposed autonomic markers, and support the establishment of consensus standards for future applications of ear thermography.

If the hypothesis is valid, there are several practical implications. In addition to emotion sensing, as described in the Introduction, measurements of ANS activity could serve several other purposes. For example, as ANS dysfunction is relevant in many clinical conditions [77], detailed assessment of SNS and PSNS activity would aid medical diagnosis and health monitoring. As ANS activity has been reported to be useful in estimating customers’ attitudes to products [78], ear temperature data may predict customers’ purchase behavior, which could assist marketers. We expect that thermal imaging of the ear would be valuable in these contexts if future validation studies are successful. This is contact-free, not susceptible to facial motion artifacts such as speaking and eating, and measurable in situations in which people wear glasses or face masks.

In conclusion, we propose a testable hypothesis that ear thermography may serve as an indicator of SNS and PSNS activity with seconds-scale time resolution. We hope that this may stimulate research interest in ear temperature.

## Data Availability

No new data were created or analyzed in this study.

## References

[1] James W. (1884). What is an emotion?. Mind.

[2] Lang P.J. (1994). The varieties of emotional experience: A meditation on James–Lange theory. Psychol. Rev..

[3] Friedman B.H. (2010). Feelings and the body: The Jamesian perspective on autonomic specificity of emotion. Biol. Psychol..

[4] Sato W. (2024). Advancements in sensors and analyses for emotion sensing. Sensors.

[5] Wehrwein E.A., Orer H.S., Barman S.M. (2016). Overview of the anatomy, physiology, and pharmacology of the autonomic nervous system. Compr. Physiol..

[6] Gershon M.D. (1999). The enteric nervous system: A second brain. Hosp. Pract..

[7] Waxenbaum J.A., Reddy V., Das J.M. (2025). Anatomy, Autonomic Nervous System. StatPearls [Internet].

[8] Benarroch E.E. (1993). The central autonomic network: Functional organization, dysfunction, and perspective. Mayo Clin. Proc..

[9] Guyenet P.G. (2006). The sympathetic control of blood pressure. Nat. Rev. Neurosci..

[10] Loewy A.D., Spyer K.M. (1990). Central Regulation of Autonomic Functions.

[11] Kreibig S.D. (2010). Autonomic nervous system activity in emotion: A review. Biol. Psychol..

[12] Boucsein W. (2011). Electrodermal Activity.

[13] Shaffer F., Ginsberg J. (2017). An overview of heart rate variability metrics and norms. Front. Public Health.

[14] Sammito S., Thielmann B., Klussmann A., Deusen A., Braumann K.M., Bockelmann I. (2024). Guideline for the application of heart rate and heart rate variability in occupational medicine and occupational health science. J. Occup. Med. Toxicol..

[15] Gates K.M., Gatzke-Kopp L.M., Sandsten M., Blandon A.Y. (2015). Estimating time-varying RSA to examine psychophysiological linkage of marital dyads. Psychophysiology.

[16] Shaffer F., Meehan Z.M., Zerr C.L. (2020). A critical review of ultra-short-term heart rate variability norms research. Front. Neurosci..

[17] Burma J., Graver S., Miutz L., Macaulay A., Copeland P., Smirl J. (2021). The validity and reliability of ultra-short-term heart rate variability parameters and the influence of physiological covariates. J. Appl. Physiol..

[18] Schafer T., Zimmermann D., Sedlmeier P. (2014). How we remember the emotional intensity of past musical experiences. Front. Psychol..

[19] Strijbosch W. (2019). From experience to memory: On the robustness of the peak-and-end rule for complex, heterogeneous experiences. Front. Psychol..

[20] Houben M. (2015). The relation between short-term emotion dynamics and psychological well-being: A meta-analysis. Psychol. Bull..

[21] Dellacherie D., Roy M., Hugueville L., Peretz I., Samson S. (2011). The effect of musical experience on emotional self-reports and psychophysiological responses to dissonance. Psychophysiology.

[22] Cannon W.B. (1915). Bodily Changes in Pain, Hunger, Fear, and Rage.

[23] Garrett J.R. (1987). The proper role of nerves in salivary secretion: A review. J. Dent. Res..

[24] Giuliano F., Rampin O. (2000). Central neural regulation of penile erection. Neurosci. Biobehav. Rev..

[25] Gross J.J., Frederickson B.L., Levenson R.W. (1994). The psychophysiology of crying. Psychophysiology.

[26] Vingerhoets A.J. (1985). The role of the parasympathetic division of the autonomic nervous system in stress and the emotions. Int. J. Psychosom..

[27] Tang B., Sato W. (2025). Ear thermal imaging for emotion sensing. Sci. Rep..

[28] Tang B., Sato W. (2026). Machine learning-based ear thermal imaging for emotion sensing. Sensors.

[29] Ioannou S., Gallese V., Merla A. (2014). Thermal infrared imaging in psychophysiology: Potentialities and limits. Psychophysiology.

[30] Kosonogov V., De Zorzi L., Honoré J., Martínez-Velázquez E.S., Nandrino J.-L., Martinez-Selva J.M., Sequeira H. (2017). Facial thermal variations: A new marker of emotional arousal. PLoS ONE.

[31] Sonkusare S., Ahmedt-Aristizabal D., Aburn M.J., Nguyen V.T., Pang T., Frydman S., Frydman S., Denman S., Fookes C., Breakspear M. (2019). Detecting changes in facial temperature induced by a sudden auditory stimulus based on deep learning-assisted face tracking. Sci. Rep..

[32] Sato W., Kochiyama T., Yoshikawa S. (2020). Physiological correlates of subjective emotional valence and arousal dynamics while viewing films. Biol. Psychol..

[33] Tang B., Sato W., Shimokawa K., Hsu C.T., Kochiyama T. (2025). Development of pixel-based facial thermal image analysis for emotion sensing. Comput. Hum. Behav. Rep..

[34] Merla A., Romani G.L. Thermal signatures of emotional arousal: A functional infrared imaging study. Proceedings of the 29th Annual International Conference of the IEEE Engineering in Medicine and Biology Society (EMBC 2007).

[35] Pavlidis I., Eberhardt N.L., Levine J. (2002). Seeing through the face of deception. Nature.

[36] Tan C.L., Knight Z.A. (2018). Regulation of body temperature by the nervous system. Neuron.

[37] Burnstock G. (2008). Non-synaptic transmission at autonomic neuroeffector junctions. Neurochem. Int..

[38] Imanishi N., Nakajima H., Aiso S. (1997). Arterial anatomy of the ear. Okajimas Folia Anat. Jpn..

[39] Cakmak Y.O., Cotofana S., Jäger C., Morawski M., Sora M.C., Werner M., Hammer N. (2018). Peri-arterial autonomic innervation of the human ear. Sci. Rep..

[40] Shireen S., Karadkhelkar V.P. (2015). Anthropometric measurements of human external ear. J. Evol. Med. Dent. Sci..

[41] Peuker E.T., Filler T.J. (2002). The nerve supply of the human auricle. Clin. Anat..

[42] Butt M.F., Albusoda A., Farmer A.D., Aziz Q. (2020). The anatomical basis for transcutaneous auricular vagus nerve stimulation. J. Anat..

[43] Rodríguez-Vázquez J.F., Verdugo-López S., Kim J.H., Hirano-Kawamoto A., Murakami G., Yamamoto M. (2025). Origin, communication and course of the vagus nerve auricular branch. Ann. Anat..

[44] Kiyokawa J., Yamaguchi K., Okada R., Maehara T., Akita K. (2014). Origin, course and distribution of nerves to the external acoustic meatus. Anat. Sci. Int..

[45] Watanabe K., Tubbs R.S., Satoh S., Zomorodi A.R., Liedtke W., Labidi M., Friedman A.H., Fukushima T. (2016). Auricular branch of vagus nerve and deep ear canal pain. World Neurosurg..

[46] Joo W. (2024). Microsurgical anatomy of the glossopharyngeal nerve. Clin. Anat..

[47] Satomi H., Yamamoto T., Ise H., Takahashi K. (1979). Identification of the inferior salivatory nucleus. Neurosci. Lett..

[48] Rezek O., Boldogkoi Z., Tombacz D., Kovago C., Gerendai I., Palkovits M., Toth I.E. (2008). Location of parotid preganglionic neurons in the inferior salivatory nucleus and their relation to the superior salivatory nucleus of rat. Neurosci. Lett..

[49] Suwabe T., Fukami H., Bradley R.M. (2008). Synaptic responses of neurons controlling the parotid and von Ebner salivary glands in rats to stimulation of the solitary nucleus and tract. J. Neurophysiol..

[50] Ishii H., Niioka T., Izumi H. (2010). Vagal visceral inputs to the nucleus tractus solitarius: Involvement in a parasympathetic reflex vasodilator pathway in the rat masseter muscle. Brain Res..

[51] Neuhuber W.L., Berthoud H.R. (2021). Functional anatomy of the vagus system—Emphasis on the somato-visceral interface. Auton. Neurosci..

[52] Izumi H., Karita K. (1992). Selective excitation of parasympathetic nerve fibers to elicit vasodilatation in cat lip. J. Auton. Nerv. Syst..

[53] Izumi H. (1999). Nervous control of blood flow in the orofacial region. Pharmacol. Ther..

[54] Clancy J.A., Mary D.A., Witte K.K., Greenwood J.P., Deuchars S.A., Deuchars J. (2014). Non-invasive vagus nerve stimulation in healthy humans reduces sympathetic nerve activity. Brain Stimul..

[55] Kaniusas E., Kampusch S., Tittgemeyer M., Panetsos F., Gines R.F., Papa M., Kiss A., Podesser B., Cassara A.M., Tanghe E. (2019). Current directions in the auricular vagus nerve stimulation I—A physiological perspective. Front. Neurosci..

[56] Cakmak Y.O. (2019). Concerning auricular vagal nerve stimulation: Occult neural networks. Front. Hum. Neurosci..

[57] Kravchik L., Ng M., Hsu N.M., VanHoy T.B. (2024). Auricular Branch of the Vagus Nerve. StatPearls [Internet].

[58] Kränzl B., Kränzl C. (1976). The role of the autonomic nervous system in trigeminal neuralgia. J. Neural Transm..

[59] Kim H.K., Chung K.M., Xing J., Kim H.Y., Youn D.H. (2024). The trigeminal sensory system and orofacial pain. Int. J. Mol. Sci..

[60] Mercante B., Ginatempo F., Manca A., Melis F., Enrico P., Deriu F. (2018). Anatomo-physiologic basis for auricular stimulation. Med. Acupunct..

[61] Ruef A.M., Levenson R.W., Coan J.A., Allen J.J.B. (2007). Continuous measurement of emotion: The affect rating dial. Handbook of Emotion Elicitation and Assessment.

[62] Friston K.J., Holmes A.P., Worsley K.J., Poline J.B., Frith C.D., Frackowiak R.S.J. (1995). Statistical parametric maps in functional imaging: A general linear approach. Hum. Brain Mapp..

[63] Lang P.J., Bradley M.M., Cuthbert B.N. (1998). Emotion, motivation, and anxiety: Brain mechanisms and psychophysiology. Biol. Psychiatry.

[64] Cacioppo J.T., Berntson G.G., Klein D.J., Clark M.S. (1992). What is an emotion? The role of somatovisceral afference. Emotion and Social Behavior.

[65] Breiman L. (2001). Random forests. Mach. Learn..

[66] He K., Zhang X., Ren S., Sun J. Deep residual learning for image recognition. Proceedings of the IEEE Conference on Computer Vision and Pattern Recognition (CVPR).

[67] Selvaraju R.R., Cogswell M., Das A., Vedantam R., Parikh D., Batra D. Grad-CAM: Visual explanations from deep networks via gradient-based localization. Proceedings of the IEEE International Conference on Computer Vision (ICCV).

[68] Lundberg S.M., Lee S.I., Guyon I., Von Luxburg U., Bengio S., Wallach H., Fergus R., Vishwanathan S.V.N., Garnett R. (2017). A unified approach to interpreting model predictions. Advances in Neural Information Processing Systems 30 (NeurIPS 2017).

[69] Goldberger J.J., Challapalli S., Tung R., Parker M.A., Kadish A.H. (2001). Relationship of heart rate variability to parasympathetic effect. Circulation.

[70] Christen S., Delachaux A., Dischl B., Golay S., Liaudet L., Feihl F., Waeber B. (2004). Dose-dependent vasodilatory effects of acetylcholine and local warming on skin microcirculation. J. Cardiovasc. Pharmacol..

[71] Droog E.J., Henricson J., Nilsson G.E., Sjoberg F. (2004). A protocol for iontophoresis of acetylcholine and sodium nitroprusside that minimises nonspecific vasodilatory effects. Microvasc. Res..

[72] Grossman P. (2024). Respiratory sinus arrhythmia (RSA), vagal tone and biobehavioral integration: Beyond parasympathetic function. Biol. Psychol..

[73] Lee S.W., Gerdes L., Tegeler C.L., Shaltout H.A., Tegeler C.H. (2014). A bihemispheric autonomic model for traumatic stress effects on health and behavior. Front. Psychol..

[74] Zygmunt A., Stanczyk J. (2010). Methods of evaluation of autonomic nervous system function. Arch. Med. Sci..

[75] Maki K.A., Goodyke M.P., Rasmussen K., Bronas U.G. (2024). An integrative literature review of heart rate variability measures to determine autonomic nervous system responsiveness using pharmacological manipulation. J. Cardiovasc. Nurs..

[76] von Wrede R., Brohl T., Rings T., Pukropski J., Helmstaedter C., Lehnertz K. (2022). Modifications of functional human brain networks by transcutaneous auricular vagus nerve stimulation: Impact of time of day. Brain Sci..

[77] Ziemssen T., Siepmann T. (2019). The investigation of the cardiovascular and sudomotor autonomic nervous system—A review. Front. Neurol..

[78] Bell L., Vogt J., Willemse C., Routledge T., Butler L.T., Sakaki M. (2018). Beyond self-report: A review of physiological and neuroscientific methods to investigate consumer behavior. Front. Psychol..

